# Rationale, Design, and Feasibility of a Prospective Multicenter Registry Study of Anthracycline-Induced Cardiotoxicity (AIC Registry)

**DOI:** 10.3390/jcm10071370

**Published:** 2021-03-27

**Authors:** Keiko Inoue, Noriko Iida, Kazuko Tajiri, Hiroko Bando, Shigeru Chiba, Nobutaka Tasaka, Kenji Nagashio, Rumi Sasamura, Hiroyuki Naito, Momoko Murata, Siqi Li, Tomoko Ishizu, Yoko Nakazawa, Ikuo Sekine, Masaki Ieda

**Affiliations:** 1 Department of Cardiology, Faculty of Medicine, University of Tsukuba, Tsukuba 305-8575, Japan; s2030351@s.tsukuba.ac.jp (K.I.); nagashio0511@gmail.com (K.N.); l.siqi@outlook.com (S.L.); tomoco@md.tsukuba.ac.jp (T.I.); mieda@md.tsukuba.ac.jp (M.I.); 2 Clinical Laboratory, University of Tsukuba Hospital, Tsukuba 305-8576, Japan; erinonchan@yahoo.co.jp (N.I.); rumi.koshizuka@gmail.com (R.S.); h.naito1031@gmail.com (H.N.); m.murata9596@gmail.com (M.M.); 3 Department of Breast and Endocrine Surgery, Faculty of Medicine, University of Tsukuba, Tsukuba 305-8575, Japan; bando@md.tsukuba.ac.jp; 4 Department of Hematology, Faculty of Medicine, University of Tsukuba, Tsukuba 305-8575, Japan; schiba-t@md.tsukuba.ac.jp; 5 Department of Obstetrics and Gynecology, Faculty of Medicine, University of Tsukuba, Tsukuba 305-8575, Japan; tsknbtk@gmail.com; 6 Department of Cardiology, Mito Kyodo General Hospital, Mito 310-0015, Japan; ponpocoyoko@hotmail.com; 7 Department of Medical Oncology, Faculty of Medicine, University of Tsukuba, Tsukuba 305-8575, Japan; isekine@md.tsukuba.ac.jp

**Keywords:** anthracycline, cardiotoxicity, cardiomyopathy, onco-cardiology, cardio-oncology, echocardiography, biomarker

## Abstract

As the number of cancer survivors increases, cardiac management in anthracycline-treated patients has become more important. We planned to conduct a prospective multicenter registry study for comprehensive echocardiographic and biomarker data collection and an evaluation of the current practice in terms of diagnosis and management of anthracycline-induced cardiotoxicity (AIC registry). To examine the feasibility of this registry study, we analyzed the 1-year follow-up data of 97 patients registered during the first year of this registry. The AIC registry was launched in July 2016. Data on echocardiographic parameters (e.g., two-and three-dimensional [(2- and 3-D) left ventricular ejection fraction (LVEF) and global longitudinal strain (GLS)) and biomarkers (e.g., troponin T and brain natriuretic peptide) were collected before anthracycline treatment, every 3 months during the first year after starting anthracycline, and every 6 months during the second year. Eighty-three patients (86%) completed a 1-year follow-up. The measurable rates of 2D LVEF, 3D LVEF, and GLS on each visit were nearly optimal (100%, 86–93%, and 84–94%, respectively). During the 1-year follow-up, 5 patients (6.0%) developed cardiotoxicity (a reduction in LVEF ≥ 10 percentage points from baseline and <55%). The AIC registry study is feasible and will be the first study to collect sizable echocardiographic and biomarker data on cardiotoxicity in Japanese patients treated with anthracycline in a real-world setting.

## 1. Introduction

Anthracyclines are among the most commonly used chemotherapeutic agents for the treatment of hematologic malignancies and solid tumors [1]. However, their effectiveness is impaired by the development of cardiotoxicity, which negatively affects patients’ quality of life and prognosis [2]. It is well established that the risk of heart failure (HF) secondary to anthracyclines is associated with cumulative exposure [3]. Therefore, the lifetime cumulative doxorubicin-equivalent exposure is limited to 400–450 mg/m^2^ in order to reduce the incidence of congestive HF to less than 5%. Recently, however, an asymptomatic decrease in the left ventricular ejection fraction (LVEF) has been frequently documented even at lower doses [4]. Thus, the incidence, time of onset, responsiveness to HF therapy, and prognosis of cardiotoxicity (symptomatic and asymptomatic LVEF decline) have not been fully elucidated.

Although the LVEF has been widely used to monitor cardiotoxicity after chemotherapy, a detectable decrease in LVEF only occurs after a considerable degree of myocardial damage. Furthermore, only 11% of patients who undergo HF treatment after a drop in LVEF showed full recovery of cardiac function [5]. Thus, it may be too late to start HF treatment when a decrease in LVEF is evident. More sensitive and accurate markers of subclinical myocardial injury are needed to enable the earlier initiation of HF treatment.

Several studies have demonstrated that modern echocardiographic techniques, such as speckle-tracking echocardiography and 3-dimensional (3D) echocardiography, and biomarkers may be useful in the early detection of cardiac injury [6,7,8,9]. However, their sample sizes were small (≤100 participants) and their follow-up periods were relatively short (≤15 months). In addition, there is limited information on the practice of cardiac imaging and biomarkers for clinical routine monitoring of patients treated with anthracyclines. 

Therefore, we planned to conduct a prospective registry study for comprehensive echocardiographic and biomarker data collection and evaluation of the current practice in terms of diagnosis and management of anthracycline-induced cardiotoxicity (AIC registry). The main objectives of the present study are as follows: (i) To investigate the incidence of anthracycline-induced cardiotoxicity (symptomatic or asymptomatic LVEF decline); (ii) to examine changes in echocardiographic parameters and biomarkers over time to define early marker(s) of cardiotoxicity; and (iii) to evaluate the response to HF treatment in patients with cardiotoxicity.

## 2. Materials and Methods

### 2.1. Design

The AIC registry study is an ongoing prospective, multicenter, observational study of anthracycline-treated patients in Ibaraki prefecture, Japan. The registry was launched in July 2016, with a set sample size of 500 patients. According to the study protocol, pre-anthracycline therapy cardiac assessment, monitoring, and management of cardiotoxicity are performed by onco-cardiology specialists.

### 2.2. Patient Eligibility

The study subjects were adult patients undergoing anthracycline-based chemotherapy. The inclusion and exclusion criteria are listed in Table 1.

### 2.3. Definition of Cardiotoxicity

Cardiotoxicity was defined as a reduction in LVEF of ≥10 percentage points from baseline and <55%, according to the Cardiac Review and Evaluation Committee criteria [10].

### 2.4. Study Protocol

**Figure 1:**
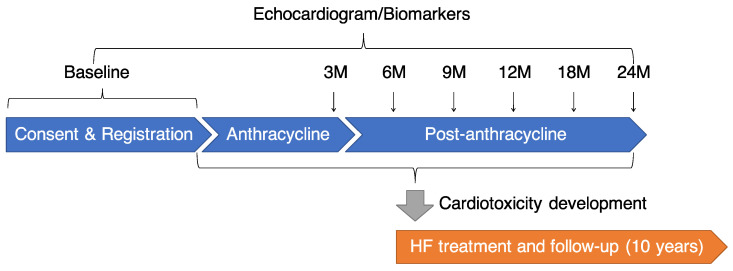
Flow chart of study. HF: heart failure, M: month.

After providing signed informed consent, patients were studied before starting anthracycline therapy, every 3 months during the first year after starting anthracycline treatment, and every 6 months during the second year, for a total of assessments studies over 2 years (Figure 1). In patients showing evidence of cardiotoxicity as defined above, treatment with an angiotensin-converting enzyme inhibitor and/or a β-blocker treatment was promptly initiated and up-titrated to the maximal tolerated dose. Additional HF treatment was provided when needed, based on the current standards of care [11]. Patients with cardiotoxicity will be studied for 10 years. During follow-up, patients were considered as responders to HF treatment when the LVEF increased up to the normal limit of 55%, as partial responders when the LVEF increased by at least 10 absolute points but did not reach the limit of 55%, and as non-responders when the LVEF increased by less than 10 absolute points and did not reach the limit of 55%.

### 2.5. Echocardiogram

All patients underwent an echocardiogram before starting anthracycline and at every visit for 2 years (Figure 1). Comprehensive echocardiographic studies for both the left and right sides of the heart were performed using a Vivid E9 or E95 ultrasound machine (GE Healthcare, Horten, Norway) according to the established guidelines [12,13]. 2D LVEF was computed from the left ventricular (LV) end-diastolic and end-systolic volumes calculated according to the bi-plane disk summation method in apical four- and two-chamber views. Strain analyses were performed using a semi-automated speckle tracking technique (AFI, EchoPAC, GE Medical Systems, Milwaukee, Brookfield, WI, USA). The three apical views were used to obtain an average global peak systolic longitudinal strain (GLS) with systole. A 3D full-volume acquisition of the LV using a matrix array transducer with the highest possible volume rate was attempted in all patients, and LV volumes and LVEF were measured offline (3DLVQ, EchoPAC, GE Medical Systems, Milwaukee, Brookfield, WI, USA). 

### 2.6. Biomarkers

Troponin T (TnT) and B-type natriuretic peptide (BNP) levels were measured at baseline and every visit during the follow-up period (Figure 1). The TnT levels were determined using a Cobas 8000 (Roche, Basel, Switzerland), while BNP levels were determined using a Lumipulse Presto II (Fujirebio Inc., Tokyo, Japan). All measurements were performed at the Tsukuba i-laboratory Limited Liability Partnership, Tsukuba, Japan.

### 2.7. Statistics

We analyzed 97 cases registered during the first year of this registry to examine the feasibility of this study. A completion rate of 1-year follow-up > 80% and a measurable rate of echocardiographic parameters > 80% were defined as the benchmarks for study feasibility. Continuous variables are presented as the mean ± standard deviation (SD), and categorical variables are presented as *n* (%). Friedman test with Bonferroni post hoc test were used to evaluate the difference in continuous variables at baseline, 3, 6, 9, and 12 months after anthracycline administration. All tests were two-tailed, and a *p*-value < 0.05 was considered significant. All statistical analyses were performed with EZR [14] for R (The R Foundation for Statistical Computing, Vienna, Austria), which is a modified version of R commander that is designed to add statistical functions frequently used in biostatistics.

## 3. Results

**Figure 2:**
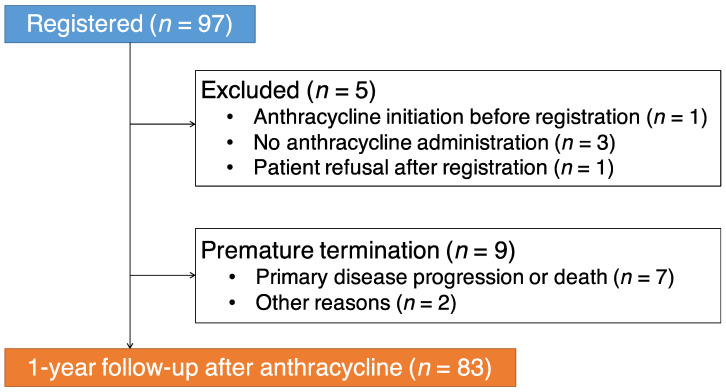
Flow of registration and follow-up.

To examine the feasibility of this registry study, we analyzed the 1-year follow-up data of 97 cases registered during the first year of this registry. Five patients were excluded at baseline for the following reasons: Anthracycline administration had already started before registration (*n* = 1), no anthracycline administration (*n* = 3), and patient refusal after registration (*n* = 1) (Figure 2). Nine patients discontinued the study within 12 months because of primary disease progression or death (*n* = 7) or other reasons (*n* = 2) (Figure 2). Relevant data of the remaining 83 patients (90.4% women; mean age, 54 ± 13 years) included in this preliminary study are reported in Table 2. During the 1-year follow-up, 5 patients (6.0%) developed cardiotoxicity (a reduction of LVEF ≥ 10% to < 55%). There was no difference in the anthracycline dose between the patients with and without cardiotoxicity (173 ± 68 vs. 200 ± 56 mg/m^2^, *p* = 0.18).

**Figure 3:**
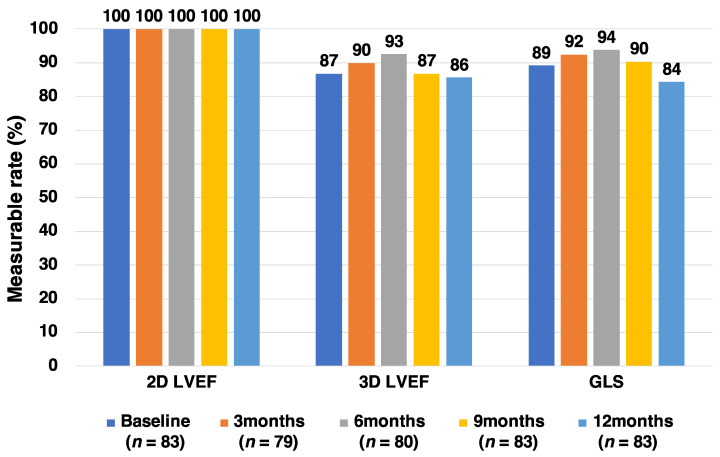
Measurable rates of 2D LVEF, 3D LVEF, and GLS.2D: Two-dimensional, 3D: Three-dimensional, GLS: Global longitudinal strain, LVEF: Left ventricular ejection fraction.

The measurable rates of the 2D LVEF, 3D LVEF, and GLS before and after anthracycline administration are shown in Figure 3. The measurable rate of 2D LVEF was very good at each time point (100%), while those of 3D LVEF and GLS were relatively poor (84–90%). The reasons for the low measurable rates of 3D LVEF and GLS were as follows: overweight, difficult postural change, left mastectomy, and left breast prosthesis.

**Figure 4:**
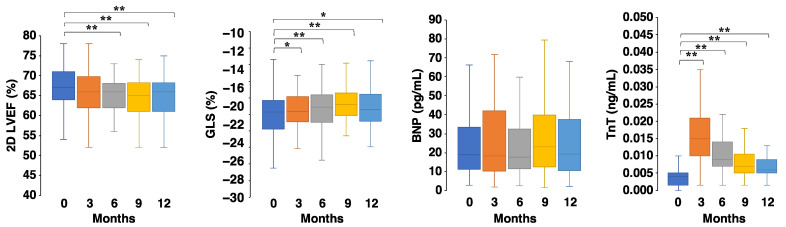
Behavior of echocardiographic parameters and biomarkers. The black line indicates the median; box, 25–75% range; whisker, 10–90% range. 2D: Two-dimensional, BNP: B-type natriuretic peptide, GLS: Global longitudinal strain, LVEF: Left ventricular ejection fraction, TnT: Troponin T. * *p* < 0.05, ** *p* < 0.01, Friedman test with Bonferroni post hoc test.

Figure 4 shows the behaviors of echocardiographic parameters and biomarkers in anthracycline-treated patients during the first year after starting anthracycline. No significant differences were observed in the BNP, while GLSs/TnTs at 3, 6, 9, and 12 months were impaired/increased compared to those at baseline.

## 4. Discussion

The AIC registry study was designed to investigate the incidence of anthracycline-induced cardiotoxicity, identify early marker(s) of cardiotoxicity, and evaluate the efficacy of HF treatment against cardiotoxicity. This is the first study to collect sizable echocardiographic and biomarker data on cardiotoxicity over time in Japanese cancer patients in a real-world setting. This study will generate important information including the predisposing factors for the development of cardiotoxicity, the rate of subclinical LV dysfunction and its transition to overt HF, the response rate to HF treatment, and the outcome of anthracycline-induced cardiotoxicity.

This preliminary analysis tested the feasibility of this registry using data from the initial 97 patients. We demonstrated a relatively lower rate of exclusion and premature termination than was initially anticipated, and 86% of the registered patients completed a 1-year follow-up (Figure 1). The incidence of cardiotoxicity during the first year after anthracycline initiation was 6.0%; this was expected as a previous observational study demonstrated that the incidence of cardiotoxicity was 9% during a median follow-up period of 5.2 years [5]. The measurable rate of 3D LVEF was suboptimal (86–93%), but similar to (or slightly better than) previously reported (66–88%) [8]. The measurable rate of GLS (84–94%) was similar to previously reported (90–91%) [8]. Overall, from the findings of this preliminary analysis, we conclude that this registry study is feasible.

In this preliminary analysis, the value of 2D LVEF decreased 6 months after anthracycline administration. However, TnT/GLS was increased/impaired 3 months after anthracycline administration. Thus, these parameters, alone or in combination, may be early markers of cardiotoxicity. We will demonstrate the clinical value of these parameters for the early diagnosis and monitoring of cardiotoxicity following completion of this registry study.

## 5. Conclusions

The AIC registry study is feasible and will be the first study to collect sizable echocardiographic and biomarker data on cardiotoxicity in Japanese patients treated with anthracycline in a real-world setting. This registry will build an evidence base that is necessary to improve future cardiac management strategies for anthracycline-treated patients.

## Figures and Tables

**Table 1:** Inclusion and exclusion criteria.

Inclusion Criteria
1. Scheduled to receive anthracyclines
2. Age ≥ 20 years
3. Oncologic life expectancy of >1 year
4. Able and willing to provide written informed consent to participate in the study
Exclusion criteria
1. Ejection fraction at baseline echo < 55%
2. Valvular stenosis or regurgitation of >moderate severity, congenital heart disease, or cardiomyopathy
3. Pregnant or lactating
4. Existing mental disorder which may affect the ability or willingness to provide informed consent
5. Considered unfit by physicians to participate in the study

**Table 2:** Baseline characteristics of the study subjects (*n* = 83).

Variable	Value
Age at administration (years)	54 ± 13
Female sex	75 (90.4)
BMI (kg/m^2^)	23.5 ± 4.3
Prior cardiovascular disease *	4 (4.8)
Diabetes mellitus	7 (8.4)
Dyslipidemia	18 (21.7)
Hypertension	16 (19.3)
Anthracycline Agents	
Epirubicin	58 (69.9)
Doxorubicin	19 (22.9)
Liposomal doxorubicin	5 (6.0)
Daunorubicin	2 (2.4)
Idarubicin	2 (2.4)
Mitoxantrone	1 (1.2)
Pirarubicin	1 (1.2)
Total cumulative dose of anthracyclines (mg/m^2^) #	198 ± 57
History of anthracyclines use	4 (4.8)
Cancer Diagnosis	
Breast cancer	58 (69.9)
Malignant lymphoma	17 (20.5)
Ovarian cancer	4 (4.8)
Leukemia	2 (2.4)
Endometrial cancer	2 (2.4)

Data are expressed as *n* (%) or mean ± SD. * Includes paroxysmal supraventricular tachycardia (*n* = 1), paroxysmal atrial fibrillation (*n* = 2), ventricular septal defect (*n* = 1). # The cumulative anthracycline dose was calculated by converting different anthracycline agents in terms of doxorubicin equivalents.

## Data Availability

The data that support the findings of this study are available from the corresponding author (K.T.), upon reasonable request.
